# Abundance and morphology of *Paleodictyon nodosum*, observed at the Clarion-Clipperton Zone

**DOI:** 10.1007/s12526-017-0636-0

**Published:** 2017-01-21

**Authors:** Jennifer M. Durden, Erik Simon-Lledo, Andrew J. Gooday, Daniel O. B. Jones

**Affiliations:** 1 National Oceanography Centre, University of Southampton Waterfront Campus, European Way, Southampton, UK; 2 Ocean and Earth Science, University of Southampton, National Oceanography Centre, University of Southampton Waterfront Campus, European Way, Southampton, UK

**Keywords:** *Paleodictyon*, Fossil, Clarion-Clipperton Zone, Graphoglyptid

## Abstract

*Paleodictyon* is an important trace fossil characterised by a regular hexagonal structure and typical of ancient deep-ocean habitats as far back as the Ordovician. It is represented in modern deep-sea settings by *Paleodictyon nodosum*, known from the Mid-Atlantic Ridge, the South Atlantic, and off eastern Australia. Here we report the occurrence of *P. nodosum* in the Clarion Clipperton Zone (CCZ), abyssal equatorial Pacific, an area characterised by polymetallic nodule fields. At the study site within the International Seabed Authority northeastern Area of Particular Environmental Interest (APEI-6), *P. nodosum* appeared as a compact, regular pattern of small circular openings on the seafloor, each pattern interpreted as reflecting the activity of an individual organism. The patterns had a mean size (maximum dimension) of 45 mm ± 16 mm SD (n = 841) and occurred at a density of 0.33 individuals m^−2^. Most (82%) were interrupted by nodules, but those that were not displayed both regular (59%) and irregular (41%) forms, the former having equal numbers of rows along the three axes (6 x 6 x 6 and 8 x 8 x 8). In both size and morphology, our *Paleodictyon* traces were more similar to the Australian examples than to those from the Mid-Atlantic Ridge.

## Introduction

**Fig. 1:**
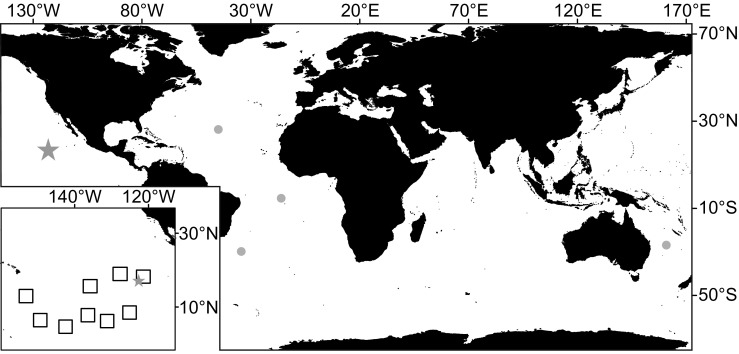
Locations globally where the modern *Paleodictyon nodosum* has been observed in deep-sea sediments (grey points), including the site of this survey (denoted as a star). A map of the APEIs of the CCZ with the study area is inset

*Paleodictyon* encompasses an important group of graphoglyptid trace fossils, because it is common and found in fossilised sediments globally. It consists of a hexagonal network of tunnels that typically appear as a pattern of holes in the seabed surface. The genus was originally described by Meneghini (1850) and is now known from ancient sediments in all seven continents (Kushlin 1982). *Paleodictyon* traces first appeared in the Lower Cambrian, migrated from shallow water to the deep sea in the early Ordovician (Buatois et al. 2009), and are particularly common, diverse, and widely distributed in Late Cretaceous and Early Tertiary strata (Seilacher 1977). Early forms were relatively simple, but the traces developed over time. In particular, trace fossils showing evidence that the hexagonal tunnels give rise to vertical shafts that intersect the seafloor as a regular array of openings first appeared in the Cretaceous (Seilacher 1977; Uchman 2003). Modern *Paleodictyon* traces resembling these were first photographed on the seabed at the Mid-Atlantic Ridge (MAR) by Rona and Merrill (1978) and have been assigned to the Eocene species *Paleodictyon nodosum* (Seilacher 1977; Ekdale 1980; Rona et al. 2009). Both the fossil and modern forms are known only from deep-sea sediments, including further observations (Fig. 1) at the MAR (Rona et al. 2009; ∼3500 m water depth), the South Atlantic (Ekdale 1980; ∼1400 and 4000 m water depth), and on the eastern margin of Australia (Przeslawski et al. 2012; 1300-2200 m water depth). Unlike fossil examples of *Paleodicyton* that are typically observed on the undersides of fine-grained turbidites (Seilacher 1977; Ehrlich 2010), the modern forms were observed on hemipelagic lutite sediment (MAR), and sandy mud of carbonate detritus (e.g. foraminifera; Australian margin).

The origin of net-like fossil *Paleodictyon* traces has proved difficult to explain (Seilacher 2007), while the organism responsible for the modern *Paleodictyon nodosum* structure has eluded discovery, despite detailed in situ submersible observations and intensive analysis of collected material (Rona et al. 2009). It has been suggested that they represent a biogenic sedimentary structure, such as a burrow (Wetzel 2000), or microbial farms for which the vertical shafts provide ventilation (Seilacher 2007; Monaco 2008). Other ideas are that the structure itself is an organism, such as a sponge (Ehrlich 2010) or a xenophyophore (Swinbanks 1982; Levin 1994). However, the detailed study of Rona et al. (2009) failed to support any of these ideas. On the sediment surface the form appears as a symmetric “honeycomb” array of holes (millimetres to a centimetre in size) arranged in three rows at an angle of 120° to each other and linked by vertical shafts to a hexagonal mesh of subsurface tunnels (Honeycutt and Plotnick 2005; Rona et al. 2009). The overall shape is hexagonal to round, with internal hexagons defined by the holes (Ehrlich 2010). Possible burrow formation mechanisms are detailed by (Wetzel 2000), but a burrowing animal would travel huge distances relative to its body length to construct such a burrow (Rona et al. 2009). The symmetry and proportional size increase have been interpreted as indicative of an organism (Rona and Merrill 1978), rather than a burrow. Some morphological variations have been observed; elongation of the pattern (Wetzel 2000) is considered to be tracemaker-controlled, while three-dimensional deformation is thought to be related to the burrowing action (Monaco 2008). Rona et al. (2009) observed some specimens to be flat, while others formed a relief above the sediment surface with a lip around the form; the former was interpreted as degraded and the latter as fresh. A “twinned” form that appeared as two overlapping individuals sharing common holes was recorded by Rona and Merrill (1978).

Here we report new observations of *Paleodictyon nodosum* from the Clarion Clipperton Zone in the Pacific Ocean, adding to the growing collection of records from the deep sea. Individual examples observed in seabed photographs collected using an autonomous underwater vehicle were used to estimate the density and to assess the morphology of in situ specimens and to compare these results with those found elsewhere in the deep sea.

## Material and methods

Data on *Paleodictyon nodosum* were derived from seabed photographs obtained during RRS *James Cook* cruise 120 to the southwest corner of the northeastern Area of Particular Environmental Interest (APEI-6) in the CCZ (17°N, 123°W; 4000 m water depth; Fig. 1) in April/May 2015 (Jones and Scientists 2015). The APEIs are areas protected from deep-sea mining (International Seabed Authority 2011). Downward-facing photographs were taken at 0.87-s intervals using a 5 MP Point Grey Research Grasshopper2 GS2-GE-50S5C camera mounted on the autonomous underwater vehicle Autosub6000 (set up as described in Morris et al. 2014). We analysed a total of 1500 non-overlapping photographs, captured between 2 and 4 m above the seabed and representing ∼2600 m^2^ of the seabed (2.4 m^2^ each, at 3.2 m altitude), calculated using photogrammetry as described by Morris et al. (2014).

Individual specimens of *P. nodosum* were identified, enumerated, and the maximum dimension was measured in randomised seabed photographs. The morphology of a subset of 120 photographed specimens was analysed in detail, including enumerating the numbers of rows of holes in the pattern, and variations in morphology were described qualitatively. Nodule characteristics were determined from 17 boxcores (seabed area 0.25 m^2^) collected in the area. Nodules found in the surface sediment (0-20 mm) were dried, counted, and their minimum and maximum dimensions were measured (Jones and Scientists 2015).

## Results and discussion

### Occurrence and density

The seabed in the APEI-6 consisted of fine-grained sediment with polymetallic nodules (mean dimensions 16 mm x 20 mm, determined from box cores) visible on the surface at a density of 338 nodules m^−2^ (standard error 53 nodules m^−2^, n = 17). A total of 841 specimens of *Paleodictyon nodosum* were observed in the photographs, equivalent to 0.33 individuals m^−2^. The density of *Paleodictyon nodosum* at APEI-6 in the CCZ was two orders of magnitude smaller than the maximum densities (45 individuals m^−2^) found at the MAR (Rona et al. 2009), and the distribution of these traces at the MAR was patchy on the scale of kilometres. *Paleodictyon* was most abundant at shallower depths (3430-3575 m) on margins of a relict hydrothermal zone on the MAR, with sparse occurrences to the end of this field (up to 4 km distance). It was absent around an active high-temperature sulphide mound, 2 km to the west of the high-abundance area. The density in the CCZ was similar to that on the shallower eastern Australian margin (0.2 individuals image^−1^), where the sediment was a sandy mud of carbonate detritus.

Modern *Paleodictyon nodosum* is thought to occur at sites with low sedimentation rates (<5 mm ka ^−1^), a characteristic of the CCZ (Khripounoff et al. 2006). It is found at the MAR, where sedimentation is estimated to be 18 mm ka^−1^ (Scott 1978 in Rona et al. 2009), but not on the Porcupine Abyssal Plain (J. Durden, pers. comm.), which has a sedimentation rate of 1.4 mm ka ^−1^ (Carvalho et al. 2011). It is also notably absent from other abyssal sites, including Station M in the eastern Pacific Ocean (4000 m water depth; Jacobsen Stout et al. 2015) and sites on the western margin of Australia (1500-4400 m water depth; Przeslawski et al. 2012). However, the association of *P. nodosum* with areas of low sedimentation may be related to their longevity. If individuals are not covered by sediment (as in the case of a turbidite) or detritus, and bioturbation is low owing to low organic matter input, then the rate of trace erasure may be low (Wheatcroft et al. 1989), and the individuals may remain visible on the seabed long after they have been abandoned (in the case of a burrow) or after the death of the organism.

### Morphology

**Fig. 2:**
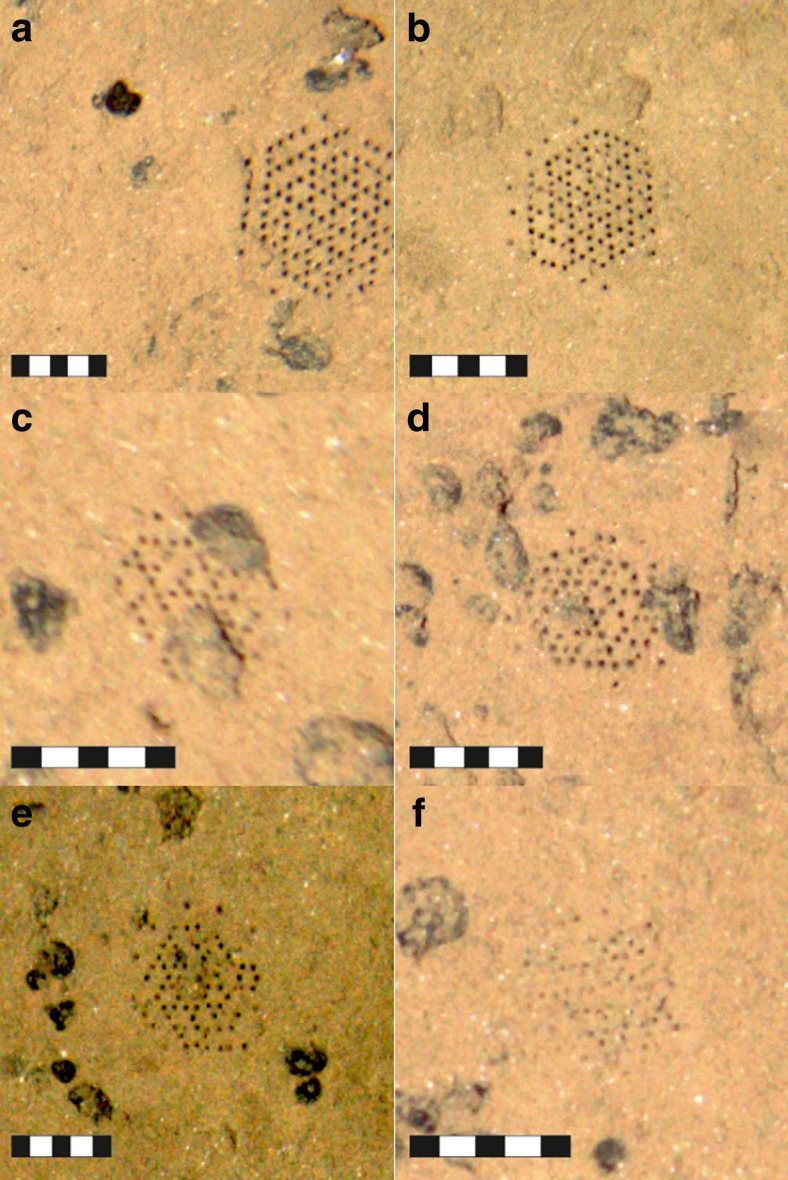
Seabed photographs showing morphological variation in *Paleodictyon nodosum* at APEI-6 at the CCZ, with scale bars showing centimetre intervals: complete specimens (**a**, **b**), specimens with surface nodules present within the pattern (**c**, **d**), specimens with a portion of the pattern missing (**e**, **f**), specimens with round holes (interpreted to be “fresh”; **a,** **b**, **e**), specimens with irregular holes (interpreted to be “degraded”; **d**, **f** )

In the APEI-6, the mean size (maximum dimension) of the *Paleodictyon nodosum* patterns was 45 mm ± 16 mm SD (n = 841). Complete forms included both regular (59%, n = 496) and irregular (41%) arrays of holes (Fig. 2). Regular forms had equal numbers of rows along the three axes (6 × 6 × 6 in 77% and 8 x 8 x 8 in 23% of complete “regular” specimens), and irregular forms generally had one axis with one row of holes fewer or more than the other two axes (e.g. 6 × 6 × 7; 7 × 7 × 8, etc.). Paleodictyon traces very similar to those observed in APEI-6 were identified during the ABYSSLLINE project in images from the UK-1 exploration claim area and the “EPIRB” area situated 250 km to the east of UK-1 (C.R. Smith, D. Amon and A. Ziegler personal communication; site locations in Amon et al. 2016). Rona and Merrill (1978) reported a similar size (∼50 mm) for *Paleodictyon* at the MAR, although the number of holes in the arrays was greater (10 × 10 × 10 to 13 × 13 × 12). The more recent investigation of Rona et al. (2009) found diameters at the MAR ranging from 24 to 75 mm in diameter (mean 50 mm), with 20 to 40 rows of holes on each axis. They also observed traces with unequal numbers of rows along the three axes, though these constituted only 1% of those analysed. The numbers of rows in *Paleodictyon nodosum* on the Australian margin was similar to that in our CCZ images (6 × 6 × 6, 7 × 7 × 7 and 8 × 8 × 8), but the overall size of the patterns found in Australia was only ∼30 mm (Dundas and Przeslawski 2009). All those illustrated in Dundas and Przeslawski (2009) contained equal numbers of rows along the three axes.

The morphology of *P. nodosum* in the APEI-6 appears to be more variable than at the MAR or on the Australian margin. Of the 120 specimens inspected in detail, only 22 (18%) were complete. The remainder were either formed around surface nodule(s), with the nodule(s) interrupting the pattern, or were missing holes in a central portion of the pattern resulting in “blank” sections in the pattern (Fig. 2). Although some nodules were found immediately below the sediment surface (<20 mm) in the box cores, covered in a thin layer of sediment, the “blank” portions of the pattern are unlikely to be attributed to the presence of such nodules, since the nodules were generally much larger than the area of missing pattern. Some specimens had very round holes, while others had irregular-shaped holes (Fig. 2). The roundness of holes could reflect how recently the structure was constructed, as the agglutination agent binding the sediment (Rona et al. 2009) may degrade over time. “Degraded” specimens were often observed to have missing portions of the pattern. At the MAR, “degraded forms” were covered in millimetres of sediment (Rona et al. 2009). In any case, it cannot be assumed that all *Paleodictyon* traces in the APEI-6 were “alive” or occupied by the maker. None of those analysed by Rona et al. (2009) yielded any sign of the organism responsible for their creation.

*Paleodictyon nodosum* has now been observed in abyssal locations in the North and South Atlantic (Rona and Merrill 1978; Ekdale 1980; Rona et al. 2009), the SW Pacific (Przeslawski et al. 2012) and the eastern equatorial Pacific (this study). The wide distribution and abundance of these traces suggests that they influence the local sedimentary structure. However, our new observations cast no light on the maker of these enigmatic traces and their function remains difficult to explain. Future imaging studies could employ time-lapse photography or video to observe the development and degradation of *Paleodictyon* and any epibenthic or bioturbation activity in the vicinity of a specimen, stereo images to examine its in situ structure, or assess co-occurrence of *Paleodictyon* to fauna or lebensspuren (life traces) in the seabed. Such studies could provide insight into the origin and ecological function of *Paleodictyon* in the deep-sea.
